# Attitudes of nursing students toward to the suicidal behavior^*^

**DOI:** 10.1590/1518-8345.2842.3116

**Published:** 2019-01-31

**Authors:** Kelly Graziani Giacchero Vedana, Ana Carolina Guidorizzi Zanetti

**Affiliations:** 1 Universidade de São Paulo, Escola de Enfermagem de Ribeirão Preto, PAHO/WHO Collaborating Centre for Nursing Research Development, Ribeirão Preto, SP, Brazil.

**Keywords:** Attitude, Suicide, Suicide, Attempted, Students, Nursing, Health Knowledge, Attitudes, Practice, Education, Nursing, Baccalaureate, Atitude, Suicídio, Tentativa de Suicídio, Estudantes de Enfermagem, Conhecimentos, Atitudes e Prática em Saúde, Bacharelado em Enfermagem, Actitud, Suicidio, Intento de Suicidio, Estudiantes de Enfermería, Conocimientos, Actitudes y Práctica en Salud, Bachillerato en Enfermería

## Abstract

**Objective:**

to investigate attitudes related to suicidal behavior and associated factors, among students in the last year of an undergraduate nursing course.

**Methods:**

a cross-sectional study with 111 nursing students from a Brazilian educational institution. The data were collected in 2017, by self-administration of a questionnaire with sociodemographic characteristics, and the Suicide Behavior Attitude Questionnaire, which were analyzed by descriptive statistics, comparison of means, and correlation tests.

**Results:**

most of the nursing students had contact with someone with suicidal behavior, but did not have education related to the subject. The most negative attitudes were associated with the female sex, lack of materials on suicide prevention, and lower self-perception of professional competence. Suicidal thoughts throughout life were associated with the contact with someone with suicidal behavior, and less moralistic/condemnatory attitudes.

**Conclusion:**

investigations and interventions are necessary for academic qualification and prevention of suicidal behavior.

## RESUMO

**Objetivo:**

investigar, entre estudantes do último ano de enfermagem, as atitudes relacionadas ao comportamento suicida e fatores associados.

**Método:**

estudo transversal com 111 estudantes de enfermagem de uma instituição de ensino brasileira. Os dados foram coletados em 2017, pela autoaplicação de um questionário sociodemográfico e do Questionário de Atitudes Frente ao Comportamento Suicida, e foram analisados por estatística descritiva, testes de comparação de média e correlação.

**Resultados:**

a maioria dos estudantes de enfermagem teve contato com alguém com comportamento suicida, mas não teve exposição educacional relacionada ao assunto. As atitudes mais negativas foram associadas ao sexo feminino: não ler materiais sobre prevenção do suicídio e menor autopercepção de competência profissional. Pensamentos suicidas foram associados ao contato com alguém com comportamento suicida e às atitudes menos moralistas/condenatórias.

**Conclusão:**

são necessárias investigações e intervenções para qualificação acadêmica e prevenção do comportamento suicida.

## RESUMEN

**Objetivo:**

investigar, entre estudiantes del último año de enfermería, las actitudes relacionadas al comportamiento suicida y factores asociados.

**Método:**

estudio transversal con 111 estudiantes de enfermería, de una institución de enseñanza brasileña. Los datos fueron recogidos en 2017 con un cuestionario sociodemográfico autoaplicado y con el Cuestionario de Actitudes Frente al Comportamiento Suicida; los datos fueron analizados por medio de la estadística descriptiva, con pruebas de comparación de medias y correlación.

**Resultados:**

la mayoría de los estudiantes de enfermería tuvo contacto con alguien que tenía comportamiento suicida, pero no tuvo exposición educacional relacionada al asunto. Las actitudes más negativas fueron asociadas a: sexo femenino, no leer materiales sobre prevención del suicidio y menor autopercepción de competencia profesional. Los pensamientos suicidas a lo largo de la vida fueron asociados al contacto con alguien con comportamiento suicida y a actitudes menos moralistas y/o de condenación.

**Conclusión:**

son necesarias nuevas investigaciones e intervenciones para la calificación académica y prevención del comportamiento suicida.

## Introduction

Suicide is considered a serious global issue that needs to be prioritized by public policies and health agendas. One death per suicide is estimated every 40 seconds, and one attempt every two or three seconds, with about 75% of suicide deaths occurring in low- and middle-income countries. Brazil occupies the eighth position in suicide numbers in the Americas. Most deaths from suicide are considered avoidable, but the topic is complex, stigmatized, and insufficiently understood^1^.

Nurses have an important role in suicide prevention ^2^ ^-^ ^4^. However, they often do not perceive themselves sufficiently prepared for providing this care ^3^. The literature has also shown association between negative attitudes related to suicide, unprepared professionals, stigma, discrimination, and inadequate quality of care^5^. However, knowledge about these issues is still limited^6^, especially among nursing students.

Only one Brazilian study was found that investigated the association between exposure to different educational strategies and attitudes related to suicide among nursing undergraduates^7^. Such attitudes were associated with: sex; having attended psychiatric nursing classes, suicide class, or laboratory; reading specific material on suicide; and suicidal thoughts throughout life^7^. This paper presents as a differential, a sample using the students in the last year of nursing undergraduation course, a period that is characterized by concluding the academic education, and by recent exposure to different knowledge addressed in the undergraduate course. Therefore, the objective of this study was to investigate attitudes related to suicidal behavior, and associated factors, among undergraduate students in the last year of the nursing school.

## Methods

This was a cross-sectional study with a quantitative approach. The hypothesis established was that attitudes related to suicidal behavior are associated with sex, age, exposure to different educational strategies (classes, laboratory, and scientific events), previous reading about suicide, and personal experiences (contact with someone who tried to commit suicide and suicidal thoughts).

Attitude is defined as the response to a stimulus that involves cognitive, affective, and behavioral components, extending to all aspects of intelligence and behavior. It is an interior disposition or propensity to action that affects the choice of action, or conduct to be adopted^8^. In the evaluation of professional attitudes related to suicidal behavior, important domains must be examined: self-perception of professional competence, negative feelings (anger, detachment, and impotence) toward the person with suicidal behavior and moralistic or condemnatory attitudes toward suicidal behavior^9^. 

This study was conducted in 2017, at a public institution of higher education, located in the interior of the state of São Paulo-SP, Brazil. The convenience sample of 111 undergraduate nursing students included all eligible students who met the selection criteria, to include as many participants as possible. The population of students enrolled in the last year of the undergraduate nursing course was eligible. Students not present during the data collection period were excluded.

Initially, the research team asked the institution for a list of students meeting the inclusion criteria. A non-faculty research team member obtained the authorization from the faculty members, who taught classes for the group of the students. In a previously agreed class period, the students were oriented about the study and ethical aspects, invited to participate in the research, signed the Terms of Free and Informed Consent Form, and received the instruments for self-completion. Verbal communication was not allowed among the students during the self-administration of the instruments

A socio-demographic questionnaire was developed by the research team with questions about sex (female or male), age, exposure to different educational strategies (suicide classes/laboratory, and suicide events or lectures), and prior reading material on suicide, previous contact with someone who attempted suicide, and an open-ended question that requested a word to define suicide. The mean time for self-administration of the instrument was 15 minutes.

The Suicide Behavior Attitude Questionnaire (SBAQ) was used to assess the attitudes of health professionals and students in cognitive, affective, and behavioral components. This is the only instrument available in Portuguese and validated in Brazil^9^. The SBAQ contains 21 statements, followed by a 10 cm solid line ranging from “strongly disagree” on one end to “strongly agree” on the other^9^. Respondents indicated the point on each line that best reflected their opinions, feelings, or reactions. The score on each SBAQ item was defined by the point of intersection between the continuous line of the instrument and the line drawn by the participant. The scores were calculated in centimeters, and the values transferred to the database with one decimal.

The authors of the instrument recommend that items be analyzed individually, or grouped into three factors. Scores on each of the three factors can range from 0 to 30 points. Factor 1 indicates “negative feelings toward the patient,” and higher scores for this factor indicate a greater presence of negative feelings. Factor 2 refers to the “self-perception of professional competence” of the interviewees, and a higher score on this factor indicates that professionals present more self-confidence in dealing with individuals with suicidal behavior. Factor 3 is defined as the “right to suicide”, and a higher score on this factor represents a less “moralistic/condemnatory” attitude. The study that developed SBAQ demonstrated good psychometric properties^9^.

After self-administration of the instruments, the data were encoded and double-entered into a structured database with a spreadsheet format in the Microsoft Excel program. Coding or typing errors were checked, compared, and corrected. The data were transferred and analyzed in the SPSS program, version 21.5^10^.

The sociodemographic, educational, and attitudinal variables related to suicidal behavior were presented using descriptive statistics. The Shapiro-Wilk normality test was applied to the subgroups to drive the option for parametric or non-parametric tests. Then, a correlation test (between continuous numerical variables) and tests of comparison of means in hypotheses related to categorical variables was used to investigate the hypotheses formulated in the study. The level of significance was established at p<0.05.

## Results

A total of 111 nursing students participated in the study. The ages ranged from 20 to 39 years, with a mean of 22.6 years. The participants’ sociodemographic and educational characteristics are presented in Table 1.

**Table 1:** Sociodemographic and educational characteristics of the nursing students (n=111), 2017

**Variable**	**N**	**%**
**Sex**		
**Female**	**96**	**86.5**
**Male**	**15**	**13.5**
**Attended mental health classes**		
**Yes**	**102**	**91.9**
**No**	**6**	**5.4**
*missing*	**3**	**2.7**
**Attended suicide prevention classes**		
**Yes**	**38**	**34.2**
**No**	**73**	**65.8**
**Participated in suicide prevention events**		
**Yes**	**31**	**27,9**
**No**	**80**	**72,1**
**Reading material on suicide prevention**		
**Yes**	**25**	**22.5**
**No**	**86**	**77.5**
**Previous contact with someone at risk of suicide**		
**Yes**	**73**	**65.8**
**No**	**38**	**34.2**

The highest scores on the SBAQ were obtained in Factor 3, “right to suicide”, and the lowest scores were obtained on Factor 1, “negative feelings”, which indicate, respectively, more comprehensive and less positive attitudes of the students in relation to the person with suicidal behavior (Table 2).

**Table 2:** Age, scores on SBAQ* factors, and suicidal thoughts throughout life (n = 111), 2017

**Variable**	**Mean**	**Standard deviation**	**Median**	**Minimum**	**Maximum**
**Age**	**22.6**	**2.4**	**22.1**	**20.1**	**39.4**
**1-Negative feeling (0 - 30)**	**9.3**	**5.7**	**9.6**	**0.0**	**24.9**
**2- Self-perception of professional competence (0 - 30)**	**12.5**	**6.0**	**11.6**	**0.7**	**30.0**
**3- Right to suicide (0 - 30)**	**16.7**	**6.7**	**16.7**	**0.1**	**30.0**
**Suicidal thoughts (0 - 10)**	**2.4**	**3.3**	**0.2**	**0.0**	**10.0**

* SBAQ - Suicide Behavior Attitude Questionnaire

The students were asked to indicate a word representing suicidal behavior. The terms were grouped, and most students associated suicidal behavior with mental distress or despair/hopelessness (61.2%) (Table 3).

**Table 3:** Terms used by nursing students to represent suicidal behavior (n = 111), 2017

**Variable**	**N**	**%**
**Mental distress**	**42**	**37.8**
**Despair/hopelessness**	**26**	**23.4**
**Dead/end**	**20**	**18.0**
**Escape/exit/freedom**	**10**	**9.0**
**Judgment (courage, cowardice, irresponsibility)**	**3**	**2.7**
**Other**	**10**	**9.0**

Comparison tests of means and correlation tests were used to assess the hypotheses that attitudes related to suicidal behavior would be associated with sex, age, exposure to different educational strategies (classes, laboratory and scientific events), previous reading material about suicide, and personal experiences (contact with someone who attempted suicide and suicidal thoughts), as provided in Tables 4 and 5.

**Table 4:** Sociodemographic characteristics and academic education according to SBAQ* scores (n = 111), 2017

**Variables**	**1- Negative feelings**		**2- Self-perception of professional competence**		**3- Right to suicide**		**Suicidal thoughts**	
	**M** ^**†**^ **(SE** ^**‡**^ **)**	**p** ^**§**^	**M** ^**†**^ **(SE** ^**‡**^ **)**	**p** ^**§**^	**M** ^**†**^ **(SE** ^**‡**^ **)**	**p** ^**§**^	**M** ^**†**^ **(SE** ^**‡**^ **)**	**p** ^**§**^
**Sex**								
**Female**	**10.0(5.6)**	**.001**	**11.6(5.5)**	**.400**	**16.2(6.7)**	**.998**	**2.4 (3.3)**	**1.00**
**Male**	**4.8(4.4)**		**17.9(6.4)**		**19.8(6.5)**		**2.4 (3.4)**	
**Classes**								
**Yes**	**9.9(5.2)**	**.366**	**13.3(5.0)**	**.137**	**15.3(6.2)**	**.626**	**2.4 (3.6)**	**.640**
**No**	**9.0(6.0)**		**12.0(6.4)**		**17.5(6.9)**		**2.4 (3.2)**	
**Events**								
**Yes**	**9.9 (5.8)**	**.460**	**12.7(6.4)**	**.489**	**17.6(6.3)**	**.564**	**2.6 (3.5)**	**.535**
**No**	**9.1(5.7)**		**12.4(5.9)**		**16.4(6.9)**		**2.3 (3.3)**	
**Contact**								
**Yes**	**9.6(5.6)**	**.372**	**12.8(5.8)**	**.701**	**16.6(6.6)**	**.890**	**3.1 (3.6)**	**<.001**
**No**	**8.8(5.9)**		**11.9(6.3)**		**16.9(7.1)**		**1.0 (2.2)**	
**Reading**								
**Yes**	**7.1(5.3)**	**.033**	**14.1(6.5)**	**.504**	**19.4(6.2)**	**.693**	**2.8 (3.9)**	**.786**
**No**	**9.9(5.7)**		**12.0(5.8)**		**15.9(6.7)**		**2.2 (3.2)**	

*SBAQ - Suicide Behavior Attitude Questionnaire; ^†^M - mean; ^‡^SE - standard error; ^§^p - p value

**Table 5:** Correlation between scores obtained on SBAQ* factors, suicidal thoughts throughout life, and age (n = 111), 2017

**Variables**	**1- Negative feelings**	**2- Self-perception of professional competence**	**3- Right to suicide**	**Suicidal thoughts**
	**r** ^**†**^ **(p value)**	**r** ^**†**^ **(p value)**	**r** ^**†**^ **(p value)**	**r** ^**†**^ **(p value)**
**1-Negative feelings**	**1**			
**2- Self-perception of professional competence**	**-0.326 (<.001)**	**1**		
**3- Right to suicide**	**-0.133 (.165)**	**-0.155 (.105)**	**1**	
**Suicidal thoughts**	**0.069 (.473)**	**-0.028 (.771)**	**0.196 (.040)**	**1**
**Age**	**-0.105 (.287)**	**0.087 (.376)**	**0.064 (.517)**	**0.119 (.229)**

*SBAQ - Suicide Behavior Attitude Questionnaire; ^†^r - Spearman coefficient

The SBAQ Factor 1 scores were higher in women and among individuals who had not read materials related to suicidal behavior prevention, indicating that people with such characteristics exhibited more negative attitudes. Factor 2 and 3 were not associated with sociodemographic and educational factors. Suicidal thoughts throughout life were more frequent among individuals that reported having had contact with someone with suicidal behavior (Table 4).

The Spearman correlation test was used to measure associations between age, suicidal thoughts, and scores on the SBAQ factors. Factor 1 showed weak negative correlations with Factor 2, which indicates that negative feelings towards the person with suicidal behavior were more intense when the students had lower self-perception of professional competence. Factor 3 presented weak positive correlations with suicidal thoughts throughout life, revealing that students who have previously thought of suicide have more understanding, and less moralistic/condemnatory attitudes, towards people with suicidal behavior (Table 5).

## Discussion

The majority of nursing students attended classes on mental health, and had contact with someone at risk for suicide. However, most did not attend classes, scientific events, courses or lectures on suicide prevention, and did not read materials on the subject. The literature reveals that Brazilian nursing students have low educational exposure related to suicide^7^, do not always read about suicide prevention on their own initiative, and prioritize revising subjects discussed in the undergraduate course tests^11^. It is important to discuss the approach to suicide prevention in a systematic way in undergraduate courses, as this is a frequent and impacting issue found in society^12^ ^-^ ^13^.

The low educational exposure related to suicide was also identified among nursing professionals of Brazilian emergency services, with the majority having experience or education related to mental health^2^. This situation contrasts with results from Spain, where most of the nurses had training (specialization in mental health or courses lasting more than 30 hours)^12^. Professionals often also feel unprepared for managing suicidal behavior ^2^, and express the need for additional training^3^. In this sense, it is important to investigate disparities related to educational exposure on suicide prevention in some contexts, despite the relevance of this topic.

Qualified education and support for students and health professionals is important for improving suicide prevention^4^ and is associated with more favorable attitudes related to suicidal behavior^13^ ^-^ ^17^. In the present study, only the reading materials on suicide were associated with better attitudes, which may be related to the size of the sample, the characteristics of the training strategies accessible to the students investigated, or the characteristics and individual experiences of those who are interested to read on the topic.

Literature presented a variety of outcomes in relation to attitudes about suicidal behavior and sociodemographic factors^18^. In this study, an association was identified only between the female sex and the most negative attitudes (anger, detachment and powerlessness) related to the individual with suicidal behavior. This is a subject that requires further investigation. However, a Brazilian study with nursing students revealed that a greater distance may be considered as an alternative for self-protection against emotional overload related to contact with suicidal behavior^11^.

The highest and lowest SBAQ scores obtained were related, respectively, to the more comprehensive and less negative attitudes toward the individual with suicidal behavior. This same pattern was observed in a sample of Brazilian nursing students,^7^ and contrasts with studies conducted with health professionals, in which more negative attitudes^12^, moralististic attitudes^2^, and less understanding and empathy with individual with suicidal behavior were predominant^14^. 

The literature has revealed that suicidal behavior is considered reprehensible, optional, surrounded by negative attitudes, misunderstanding, and considered a transgression because it is in conflict with the principles of life and the ethics of health professionals^3^ ^,^ ^11^ ^,^ ^19^ ^-^ ^21^. Contradicting these findings^3^ ^,^ ^11^ ^,^ ^19^ ^-^ ^21^, the main terms used in this study to represent suicidal behavior were associated with mental distress, despair, or hopelessness. The empathic understanding and attitude towards the individual with suicidal behavior seem to be important conditions for prevention, and need to be addressed in the health professionals’ education^21^.

Suicidal thoughts throughout life were more frequent among individuals who had contact with someone with suicidal behavior, which may be related to the phenomenon of contagion^22^. It is important to talk carefully about suicide with students with professional and academic experiences related to the subject, and also to investigate suicide risk in a detailed manner among potentially vulnerable students, as suicidal thoughts are relatively common throughout life^23^ ^-^ ^24^, and the risk of suicide tends to be fluctuating^25^ ^-^ ^26^. Other Brazilian research conducted with nursing students did not find associations between prior contact with individual at risk for suicide and attitudes related to suicide^8^. However, the literature shows that, in the university population, the contact with suicide cases can facilitate the occurrence of suicidal thoughts, fears, worries and feelings of vulnerability^27^.

Studies suggest that negative feelings and attitudes toward suicidal behavior are associated with a lack of professional preparation, and may impair the quality of care^5^ ^-^ ^6^. These results corroborate with the results of this investigation, in which negative feelings towards the individual with suicidal behavior were more intense when the students had lower self-perception of professional competence.

One limitation of this research is the cross-sectional design and the fact that it is restricted to a single educational institution of a limited geographic territory. Another limitation is the use of a convenience sample that included the entire eligible population, to comprise as many nursing students as possible during the data collection period. The sample size is another limitation. Future research should include a larger, and more representative samples. The study did not explore religious beliefs and may have been imprecise in assessing mental health guidance and experience (by self-report and without detailed duration). Regardless of these limitations, this is one of the few Brazilian studies associating attitudes towards suicidal behavior and educational strategies. This knowledge is relevant for planning interventions and conducting investigations related to the support, supervision, and education of nursing students.

## Conclusion

In this study, most nursing students had contact with someone at risk of suicide, attended mental health classes, but had not attended classes, scientific events, courses or lectures on suicide prevention, nor had they read materials on the subject. The students represented suicidal behavior mainly in terms associated with mental suffering, despair, or hopelessness.

We hypothesized that attitudes related to suicidal behavior would be associated with sex, age, exposure to different educational strategies (classes, laboratory and scientific events), previous reading material about suicide, and personal experiences (contact with someone who attempted suicide and suicidal thoughts). The most negative attitudes were identified among women, people who had not read suicide prevention materials, and individuals with perceived unpreparedness to deal with suicidal behavior. Suicidal thoughts throughout life were more frequent among people who had contact with someone with suicidal behavior, and students who previously had thought about suicide showed more understanding attitudes toward suicidal behavior. Further studies should investigate these variables in different contexts. In addition, investigations are needed to develop and investigate different strategies for academic qualification and prevention of suicidal behavior.

Previous contact with someone at risk of suicide was associated with suicidal thoughts throughout life, but not with better attitudes related to suicidal behavior, suggesting that contact with the suicidal person could be mediating, and complemented by other strategies for support, supervision, and education. In addition, it is important to monitor the students’ mental health for the development of appropriate supportive actions.
